# Supplementary material for: Effect of Chitosan on Ruminal Fermentation and Microbial Communities, Methane Emissions, and Productive Performance of Dairy Cattle
Source: Animals (Basel). 2023 Sep 8;13(18):2861. doi: 10.3390/ani13182861 (PMC10525946; doi:10.3390/ani13182861)
Supplement: Supplementary file 1 [file animals-13-02861-s001.zip › animals-2492830-supplementary.pdf]

**Table S1.** Ingredients and chemical composition of forage basal diet and concentrate.

| Item | Concentrate | Basal Diet |
|---|---|---|
| Ingredients composition (g/kg DM) | | |
| Maize silage | | 317 |
| Grass silage | | 683 |
| Maize | 318 | |
| Soybean meal | 245 | |
| Wheat middlings | 174 | |
| Palm kernel meal | 120 | |
| Hydrogenated palm fat | 25 | |
| Malt culms | 30 | |
| Cane bagasse | 30 | |
| Rapeseed meal | 30 | |
| Sunflower meal | 10 | |
| Barley | 9 | |
| Oil palm hydrogenated fat (99%) | 6.8 | |
| [1]Cows NUTEMIX | 2 | |
| Sodium chloride | 0.2 | |
| Chemical composition (% of DM) | | |
| UFL | 1.02 | |
| DM | 90 | 24 |
| Crude ash | 6.6 | 8.8 |
| Starch | 25 | 4.0 |
| Crude protein | 19 | 12 |
| Neutral detergent fiber | 21 | 49 |
| Acid detergent fiber | 10 | 27 |
| Fat | 6.2 | |

UFL: forage unit for lactation; DM: dry matter; [1]Contained: calcium (17 g/kg), magnesium (25 g/kg), sodium (40 g/kg), phosphorus (52 g/kg), zinc (15 g/kg), manganese (14 g/kg), copper (1.7 g/kg), iodine (130 mg/kg), cobalt (39 mg/kg), selenium (2 mg/kg).

**Table S2.** Main microbial phyla of rumen samples of cows fed a conventional concentrate and a concentrate with chitosan

| **Phylum (clr_RA)** | **HF** | | **BS** | | | **Adjusted P-value** | | |
|---|---|---|---|---|---|---|---|---|
| | **CTR** | **CHI** | **CTR** | **CHI** | **SEM** | **Treat** | **Breed** | **Treat x Breed** |
| Euryarchaeota | 1.10 | 0.53 | 0.75 | 0.87 | 0.198 | 0.704 | 0.988 | 0.361 |
| Actinobacteria | 0.030 | 0.253 | 0.302 | 0.193 | 0.1323 | 0.905 | 0.988 | 0.471 |
| Bacteroidetes | 4.84 | 5.20 | 5.24 | 5.17 | 0.149 | 0.751 | 0.988 | 0.398 |
| Chloroflexi | -3.22 | -3.27 | -3.47 | -3.62 | 0.233 | 0.905 | 0.988 | 0.846 |
| Cyanobacteria | -2.57 | -1.84 | -1.86 | -2.26 | 0.360 | 0.905 | 0.988 | 0.361 |
| Elusimicrobia | -1.74 | -1.30 | -1.94 | -1.75 | 0.220 | 0.582 | 0.988 | 0.696 |
| Fibrobacteres | 1.34 | 1.77 | 1.65 | 1.71 | 0.214 | 0.704 | 0.988 | 0.593 |
| Firmicutes | 3.86 | 3.80 | 3.85 | 3.97 | 0.120 | 0.905 | 0.988 | 0.596 |
| Fusobacteria | -2.20 | -2.53 | -2.45 | -2.29 | 0.142 | 0.905 | 0.988 | 0.361 |
| Kiritimatiellaeota | 0.83 | 1.11 | 1.20 | 0.97 | 0.239 | 0.933 | 0.988 | 0.575 |
| Lentisphaerae | -0.229 | 0.096 | -0.078 | -0.272 | 0.2621 | 0.905 | 0.988 | 0.575 |
| Planctomycetes | -2.54 | -2.65 | -2.63 | -2.68 | 0.120 | 0.878 | 0.988 | 0.846 |
| Proteobacteria | 1.66 | 1.81 | 1.76 | 2.03 | 0.244 | 0.819 | 0.988 | 0.846 |
| Spirochaetes | 0.36 | 1.12 | 0.66 | 0.74 | 0.119 | 0.100 | 0.988 | 0.361 |
| Synergistetes | -2.91 | -2.68 | -2.66 | -2.85 | 0.282 | 0.933 | 0.988 | 0.596 |
| Tenericutes | -1.16 | -0.49 | -0.84 | -0.68 | 0.161 | 0.294 | 0.988 | 0.361 |
| Verrucomicrobia | -1.67 | -1.81 | -1.85 | -1.78 | 0.151 | 0.905 | 0.988 | 0.593 |
| Ascomycota | 0.37 | -0.385 | -0.084 | -0.068 | 0.2307 | 0.504 | 0.988 | 0.361 |
| Basidiomycota | -0.44 | -1.35 | -0.96 | -0.92 | 0.217 | 0.504 | 0.988 | 0.361 |
| Chytridiomycota | 1.20 | 0.36 | 0.78 | 0.80 | 0.221 | 0.504 | 0.988 | 0.361 |
| Ciliophora | 3.08 | 2.27 | 2.66 | 2.72 | 0.223 | 0.504 | 0.988 | 0.361 |

RA_clr: relative abundance CLR-transformed; HF: Holstein-fresian; BS: brown Swiss; CTR: control; CHI: chitosan; SEM: standard error of the mean; Treat: treatment; Adjust P-value: adjusted P-value using False Discovery Rate.

**Table S3.** Main microbial genera of rumen samples of cows fed a conventional concentrate and a concentrate with chitosan

| Genus (clr_RA) | HF | | BS | | | Adjusted P-value | | |
|---|---|---|---|---|---|---|---|---|
| | CTR | CHI | CTR | CHI | SEM | Treat | Breed | Treat x Breed |
| *Archaea* | | | | | | | | |
| Methanobrevibacter | 2.05 | 1.64 | 1.63 | 1.85 | 0.217 | 0.686 | 0.991 | 0.281 |
| Methanosphaera | 0.94 | 0.14 | 0.77 | 0.45 | 0.207 | 0.100 | 0.991 | 0.281 |
| UC_Methanobacteriaceae | 0.76 | 0.37 | 0.69 | 0.62 | 0.131 | 0.244 | 0.991 | 0.281 |
| UC_Methanobacteriales | 0.57 | 0.19 | 0.39 | 0.40 | 0.127 | 0.244 | 0.991 | 0.281 |
| UC_Methanobacteria | 0.138 | -0.235 | 0.008 | -0.003 | 0.1320 | 0.244 | 0.991 | 0.281 |
| *Bacteria* | | | | | | | | |
| Aeriscardovia | 1.17 | 1.48 | 1.60 | 1.33 | 0.179 | 0.953 | 0.991 | 0.569 |
| Bifidobacterium | -2.11 | -0.43 | -2.30 | -1.93 | 0.471 | 0.651 | 0.991 | 0.682 |
| UC_Bifidobacteriaceae | -0.99 | -0.68 | -0.86 | -1.12 | 0.152 | 0.953 | 0.991 | 0.569 |
| UC_Bifidobacteriales | -1.38 | -1.09 | -0.86 | -1.09 | 0.125 | 0.906 | 0.991 | 0.569 |
| UC_Actynomicetia | -0.96 | -1.05 | -0.84 | -1.03 | 0.201 | 0.801 | 0.991 | 0.935 |
| UC_Coriobacteriia | -1.69 | -1.60 | -1.50 | -1.40 | 0.202 | 0.853 | 0.991 | 0.996 |
| UC_Actinobacteria | -0.84 | -0.68 | -0.58 | -0.64 | 0.146 | 0.887 | 0.991 | 0.847 |
| Bacteroides | 1.57 | 1.84 | 1.71 | 1.71 | 0.155 | 0.801 | 0.991 | 0.814 |
| UC_Bacteroidaceae | 3.79 | 4.19 | 4.18 | 4.11 | 0.143 | 0.750 | 0.991 | 0.569 |
| Barnesiella | -1.85 | -1.07 | -1.37 | -1.41 | 0.220 | 0.676 | 0.991 | 0.569 |
| UC_Muribaculaceae | 2.20 | 2.17 | 2.03 | 2.29 | 0.264 | 0.853 | 0.991 | 0.880 |
| UC_Paludibacteraceae | 3.32 | 3.46 | 3.45 | 3.48 | 0.149 | 0.853 | 0.991 | 0.928 |
| Phocaeicola | -0.88 | -0.22 | -0.41 | -0.42 | 0.182 | 0.676 | 0.991 | 0.569 |
| Alloprevotella | -0.76 | -0.34 | -0.32 | -0.43 | 0.155 | 0.758 | 0.991 | 0.569 |
| Paraprevotella | -1.59 | -1.52 | -1.66 | -1.34 | 0.251 | 0.801 | 0.991 | 0.893 |
| Prevotella | 5.90 | 6.47 | 6.47 | 6.26 | 0.207 | 0.790 | 0.991 | 0.569 |
| UC_Prevotellaceae | 3.67 | 4.27 | 4.33 | 4.08 | 0.249 | 0.801 | 0.991 | 0.569 |
| Alistipes | -1.14 | -0.83 | -1.25 | -1.05 | 0.180 | 0.676 | 0.991 | 0.928 |
| UC_Rikenellaceae | 0.10 | 0.51 | 0.36 | 0.43 | 0.146 | 0.676 | 0.991 | 0.693 |
| UC_Salinivirgaceae | -0.79 | -0.48 | -0.68 | -0.80 | 0.079 | 0.750 | 0.991 | 0.464 |
| Parabacteroides | -1.25 | -0.87 | -1.05 | -0.96 | 0.154 | 0.676 | 0.991 | 0.814 |
| UC_Tanerellaceae | -1.85 | -1.54 | -1.90 | -1.64 | 0.155 | 0.676 | 0.991 | 0.935 |
| UC_Bacteroidales | 5.25 | 5.63 | 5.58 | 5.51 | 0.125 | 0.750 | 0.991 | 0.569 |
| UC_Bacteroidia | 2.95 | 3.37 | 3.36 | 3.21 | 0.166 | 0.801 | 0.991 | 0.569 |
| UC_Flavobacteriales | -1.18 | -1.46 | -1.16 | -1.29 | 0.117 | 0.676 | 0.991 | 0.871 |
| Chryseobacterium | -0.43 | -0.78 | -0.61 | -0.66 | 0.210 | 0.758 | 0.991 | 0.871 |
| UC_Flavobacteriia | -1.35 | -1.38 | -1.30 | -1.36 | 0.145 | 0.875 | 0.991 | 0.944 |
| UC_Bacteroidetes | 3.02 | 3.34 | 3.34 | 3.20 | 0.147 | 0.822 | 0.991 | 0.569 |
| UC_Cyanobacteria | -0.92 | -0.06 | -0.08 | -0.62 | 0.446 | 0.875 | 0.991 | 0.569 |
| UC_Elusimicrobiaceae | -1.10 | -0.37 | -1.44 | -1.28 | 0.302 | 0.676 | 0.991 | 0.814 |
| UC_Elusimicrobia | -0.51 | -0.05 | -0.56 | -0.47 | 0.277 | 0.758 | 0.991 | 0.871 |
| Fibrobacter | 2.98 | 3.54 | 3.39 | 3.36 | 0.264 | 0.758 | 0.991 | 0.722 |
| UC_Fibrobacteraceae | 0.001 | 0.527 | 0.236 | 0.211 | 0.2031 | 0.750 | 0.991 | 0.689 |
| UC_Fibrobacterales | -0.51 | -0.17 | -0.33 | -0.29 | 0.170 | 0.758 | 0.991 | 0.814 |
| UC_Fibrobacteria | -1.03 | -0.77 | -1.10 | -0.89 | 0.183 | 0.750 | 0.991 | 0.935 |

| | | | | | | | | |
|---|---|---|---|---|---|---|---|---|
| UC_Fibrobacteres | -1.58 | -1.04 | -1.20 | -1.34 | 0.130 | 0.676 | 0.991 | 0.464 |
| Bacillus | -1.94 | -1.66 | -1.72 | -1.87 | 0.242 | 0.922 | 0.991 | 0.814 |
| Staphylococcus | -1.11 | -0.32 | -1.00 | -1.04 | 0.371 | 0.758 | 0.991 | 0.722 |
| UC_Bacillales | -1.30 | -1.38 | -1.03 | -1.21 | 0.134 | 0.758 | 0.991 | 0.928 |
| Leuconostoc | -3.64 | -1.83 | -3.10 | -2.83 | 0.555 | 0.676 | 0.991 | 0.682 |
| UC_Lactobacillaceae | -2.32 | -2.41 | -2.34 | -2.56 | 0.255 | 0.847 | 0.991 | 0.935 |
| Streptococcus | -1.32 | -1.48 | -1.46 | -1.49 | 0.191 | 0.853 | 0.991 | 0.928 |
| UC_Lactobacillales | -0.70 | -0.98 | -0.77 | -0.75 | 0.169 | 0.801 | 0.991 | 0.815 |
| UC_Bacilli | 2.82 | 2.70 | 2.88 | 2.81 | 0.097 | 0.758 | 0.991 | 0.928 |
| Clostridium | 0.96 | 0.99 | 0.93 | 1.02 | 0.133 | 0.853 | 0.991 | 0.935 |
| Sarcina | -1.01 | -1.01 | -1.02 | -0.84 | 0.204 | 0.853 | 0.991 | 0.925 |
| UC_Clostridiaceae | 0.14 | 0.03 | 0.02 | 0.12 | 0.200 | 0.982 | 0.991 | 0.814 |
| Eubacterium | 0.93 | 1.35 | 1.43 | 1.30 | 0.174 | 0.801 | 0.991 | 0.569 |
| UC_Eubacteriaceae | -0.38 | -0.66 | -0.40 | -0.50 | 0.136 | 0.676 | 0.991 | 0.871 |
| Agathobacter | -1.74 | -1.30 | -1.34 | -1.30 | 0.354 | 0.807 | 0.991 | 0.880 |
| Blautia | -1.39 | -1.52 | -1.89 | -1.73 | 0.193 | 0.953 | 0.991 | 0.871 |
| Butyrivibrio | 1.83 | 2.01 | 1.88 | 2.00 | 0.178 | 0.790 | 0.991 | 0.935 |
| Coprococcus | -1.81 | -1.51 | -2.32 | -1.71 | 0.220 | 0.651 | 0.991 | 0.871 |
| Oribacterium | -0.518 | 0.164 | -0.024 | -0.047 | 0.209 | 0.676 | 0.991 | 0.569 |
| Pseudobutyrivibrio | 0.34 | 0.67 | 0.64 | 0.74 | 0.212 | 0.758 | 0.991 | 0.880 |
| Roseburia | -0.74 | 0.33 | -0.50 | -0.11 | 0.359 | 0.651 | 0.991 | 0.814 |
| Stomatobaculum | -0.398 | -0.022 | -0.580 | -0.662 | 0.244 | 0.847 | 0.991 | 0.814 |
| UC_Lachnospiraceae | 3.16 | 3.31 | 3.30 | 3.34 | 0.098 | 0.758 | 0.991 | 0.878 |
| Ruminiclostridium | -1.77 | -2.72 | -1.46 | -1.72 | 0.319 | 0.676 | 0.991 | 0.748 |
| Ruminococcus | 2.15 | 2.75 | 2.58 | 2.62 | 0.139 | 0.651 | 0.991 | 0.569 |
| UC_Oscillospiraceae | 3.13 | 3.26 | 3.14 | 3.23 | 0.150 | 0.801 | 0.991 | 0.935 |
| UC_Eubacteriales | 4.01 | 4.00 | 4.01 | 4.07 | 0.097 | 0.887 | 0.991 | 0.928 |
| UC_Clostridia | 4.11 | 3.91 | 4.03 | 3.99 | 0.104 | 0.758 | 0.991 | 0.847 |
| Sharpea | -3.78 | -2.15 | -3.13 | -2.32 | 0.773 | 0.676 | 0.991 | 0.880 |
| UC_Erysipelotrichaceae | 0.68 | 1.18 | 0.81 | 0.99 | 0.120 | 0.464 | 0.991 | 0.689 |
| UC_Erysipelotrichales | -0.76 | -0.17 | -0.56 | -0.14 | 0.263 | 0.676 | 0.991 | 0.928 |
| UC_Erysipelotrichia | -0.72 | -0.64 | -0.74 | -0.59 | 0.107 | 0.758 | 0.991 | 0.928 |
| Succiniclasticum | 1.54 | 1.49 | 1.58 | 1.64 | 0.242 | 0.982 | 0.991 | 0.935 |
| Acidaminococcaceae | 1.38 | 1.36 | 1.45 | 1.53 | 0.200 | 0.946 | 0.991 | 0.928 |
| Quinella | -0.83 | -1.31 | -0.98 | -1.14 | 0.156 | 0.651 | 0.991 | 0.791 |
| Anaerovibrio | -0.035 | 1.110 | 0.655 | 0.685 | 0.2871 | 0.651 | 0.991 | 0.569 |
| Schwartzia | -0.003 | 0.376 | 0.488 | 0.580 | 0.1823 | 0.750 | 0.991 | 0.847 |
| Selenomonas | 0.79 | 1.85 | 1.63 | 1.58 | 0.312 | 0.676 | 0.991 | 0.569 |
| UC_Selenomonadaceae | -0.595 | 0.094 | -0.074 | -0.102 | 0.2252 | 0.676 | 0.991 | 0.569 |
| UC_Selenomonadales | -1.41 | -0.92 | -1.16 | -1.16 | 0.208 | 0.750 | 0.991 | 0.693 |
| UC_Negativicutes | -0.34 | -0.26 | -0.17 | -0.18 | 0.089 | 0.868 | 0.991 | 0.885 |
| Megasphaera | -3.78 | -3.87 | -3.36 | -3.06 | 0.321 | 0.868 | 0.991 | 0.878 |
| UC_Firmicutes | 3.75 | 3.74 | 3.77 | 3.80 | 0.055 | 0.940 | 0.991 | 0.925 |
| Fusobacterium | -1.69 | -1.86 | -1.71 | -1.68 | 0.165 | 0.853 | 0.991 | 0.878 |
| UC_Kiritimatiellae | 2.54 | 2.95 | 2.99 | 2.67 | 0.301 | 0.946 | 0.991 | 0.693 |
| UC_Kiritimatiellaeota | -0.72 | -0.56 | -0.51 | -0.76 | 0.239 | 0.940 | 0.991 | 0.814 |
| UC_Lentisphaeria | -0.37 | 0.15 | -0.21 | -0.34 | 0.275 | 0.801 | 0.991 | 0.693 |
| UC_Victivallales | 0.313 | 0.576 | 0.333 | 0.017 | 0.443 | 0.979 | 0.991 | 0.871 |

| | | | | | | | | |
|---|---|---|---|---|---|---|---|---|
| UC_Lentisphaerae | 0.89 | 1.37 | 1.23 | 0.89 | 0.321 | 0.940 | 0.991 | 0.693 |
| UC_Planctomycetes | -1.63 | -1.99 | -1.80 | -1.94 | 0.179 | 0.676 | 0.991 | 0.878 |
| Acetobacter | -1.026 | 0.010 | -0.055 | -0.238 | 0.495 | 0.800 | 0.991 | 0.693 |
| UC_Alphaproteobacteria | 0.55 | 1.29 | 1.00 | 0.77 | 0.224 | 0.758 | 0.991 | 0.569 |
| UC_Betaproteobacteria | -1.90 | -1.32 | -1.94 | -1.76 | 0.274 | 0.676 | 0.991 | 0.871 |
| Desulfovibrio | -0.82 | -1.00 | -0.96 | -0.84 | 0.245 | 0.953 | 0.991 | 0.878 |
| Ruminobacter | 0.020 | 0.483 | 0.229 | 0.440 | 0.602 | 0.853 | 0.991 | 0.935 |
| Succinomonas | 0.11 | 0.39 | 0.41 | 0.69 | 0.405 | 0.801 | 0.991 | 0.996 |
| Succinivibrio | 0.061 | 1.297 | 0.400 | 0.559 | 0.4146 | 0.676 | 0.991 | 0.693 |
| UC_Succinivibrionaceae | 2.19 | 2.34 | 2.46 | 2.75 | 0.398 | 0.853 | 0.991 | 0.935 |
| UC_Aeromonadales | -0.392 | 0.322 | -0.308 | 0.066 | 0.3909 | 0.676 | 0.991 | 0.925 |
| Escherichia | -1.43 | -1.56 | -1.15 | -1.30 | 0.275 | 0.853 | 0.991 | 0.987 |
| Klebsiella | 0.86 | 0.40 | 0.52 | 0.51 | 0.093 | 0.651 | 0.991 | 0.565 |
| UC_Enterobacteriaceae | -0.10 | -0.50 | -0.43 | -0.28 | 0.174 | 0.801 | 0.991 | 0.569 |
| UC_Enterobacterales | -0.065 | -0.623 | -0.526 | -0.213 | 0.1693 | 0.801 | 0.991 | 0.464 |
| Acinetobacter | -0.75 | -0.65 | -0.69 | -0.77 | 0.269 | 0.982 | 0.991 | 0.928 |
| Pseudomonas | -1.96 | -1.64 | -2.09 | -1.89 | 0.326 | 0.801 | 0.991 | 0.935 |
| UC_Gammaproteobacteria | 0.86 | 0.88 | 0.86 | 1.13 | 0.281 | 0.853 | 0.991 | 0.925 |
| Vibrio | -2.20 | -1.76 | -1.99 | -1.73 | 0.318 | 0.758 | 0.991 | 0.928 |
| UC_Proteobacteria | 1.21 | 1.31 | 1.21 | 1.26 | 0.148 | 0.853 | 0.991 | 0.935 |
| Treponema | 1.48 | 2.54 | 1.96 | 1.96 | 0.171 | 0.425 | 0.991 | 0.386 |
| UC_Spirochaetaceae | 0.064 | 0.940 | 0.426 | 0.428 | 0.1637 | 0.533 | 0.991 | 0.464 |
| UC_Spirochaetales | 0.10 | 0.85 | 0.42 | 0.38 | 0.106 | 0.406 | 0.991 | 0.348 |
| UC_Spirochaetia | -0.197 | 0.069 | -0.163 | -0.085 | 0.1451 | 0.750 | 0.991 | 0.871 |
| UC_Spirochaetes | -0.46 | -0.18 | -0.38 | -0.49 | 0.118 | 0.801 | 0.991 | 0.569 |
| Pyramidobacter | -2.11 | -1.51 | -1.52 | -1.78 | 0.361 | 0.853 | 0.991 | 0.693 |
| UC_Acholeplasmatales | -1.48 | -0.79 | -0.99 | -1-10 | 0.200 | 0.676 | 0.991 | 0.569 |
| Anaeroplasma | -0.85 | 0.57 | -0.19 | 0.12 | 0.175 | 0.046 | 0.991 | 0.386 |
| Mycoplasma | -2.08 | -1.58 | -1.61 | -1.87 | 0.314 | 0.868 | 0.991 | 0.693 |
| UC_Mollicutes | -1.21 | -0.74 | -1.00 | -0.92 | 0.130 | 0.651 | 0.991 | 0.587 |
| UC_Tenericutes | -1.18 | -0.79 | -0.94 | -0.98 | 0.169 | 0.758 | 0.991 | 0.569 |
| UC_Opitutae | -1.68 | -1.55 | -2.09 | -1.92 | 0.312 | 0.853 | 0.991 | 0.976 |
| UC_Verrucomicrobia | -0.41 | -0.33 | -0.37 | -0.39 | 0.138 | 0.940 | 0.991 | 0.928 |
| *Eukaryota* | | | | | | | | |
| Sacharomyces | -1.28 | -1.84 | -1.67 | -1.58 | 0.237 | 0.408 | 0.991 | 0.239 |
| UC_Sacharomycetacceae | -1.22 | -2.10 | -1.88 | -1.97 | 0.194 | 0.133 | 0.991 | 0.207 |
| UC_Sacharomycetales | -0.81 | -1.19 | -1.30 | -1.21 | 0.231 | 0.582 | 0.991 | 0.370 |
| UC_Sordariomycetes | -1.59 | -2.14 | -1.89 | -1.93 | 0.266 | 0.372 | 0.991 | 0.382 |
| UC_Ascomycota | 0.88 | 0.25 | 0.48 | 0.42 | 0.181 | 0.134 | 0.991 | 0.208 |
| UC_Agarycomycetes | -0.18 | -0.83 | -0.45 | -0.56 | 0.184 | 0.134 | 0.991 | 0.217 |
| UC_Basidiomycota | -0.47 | -1.29 | -1.16 | -0.92 | 0.180 | 0.189 | 0.991 | 0.207 |
| Anaeromyces | 0.71 | -0.046 | 0.27 | 0.23 | 0.175 | 0.133 | 0.991 | 0.207 |
| Neocallimastix | 1.93 | 1.26 | 1.64 | 1.51 | 0.163 | 0.133 | 0.991 | 0.208 |
| Piromyces | 1.25 | 0.45 | 0.87 | 0.77 | 0.170 | 0.133 | 0.991 | 0.207 |
| UC_Neocallimastigaceae | 1.73 | 1.06 | 1.40 | 1.37 | 0.179 | 0.134 | 0.991 | 0.207 |
| Nyctotherus | -1.63 | -2.28 | -2.41 | -2.15 | 0.260 | 0.518 | 0.991 | 0.207 |
| Stentor | 2.68 | 1.95 | 2.31 | 2.27 | 0.168 | 0.133 | 0.991 | 0.207 |
| Entodinium | 2.48 | 1.79 | 2.05 | 2.05 | 0.162 | 0.133 | 0.991 | 0.207 |

| | | | | | | | | |
|---|---|---|---|---|---|---|---|---|
| Epidinium | -0.51 | -3.04 | -1.68 | -1.98 | 0.565 | 0.133 | 0.991 | 0.207 |
| Eudiplodinium | -1.51 | -1.68 | -1.45 | -1.40 | 0.228 | 0.804 | 0.991 | 0.645 |
| Polyplastron | -0.88 | -2.34 | -1.91 | -1.53 | 0.527 | 0.397 | 0.991 | 0.207 |
| Isotricha | -0.51 | -0.98 | -0.79 | -0.76 | 0.185 | 0.356 | 0.991 | 0.239 |
| Ichthyophthirius | 2.54 | 1.73 | 2.03 | 2.07 | 0.181 | 0.133 | 0.991 | 0.207 |
| Tetrahymena | 1.67 | 1.06 | 1.45 | 1.34 | 0.163 | 0.133 | 0.991 | 0.208 |
| UC_Hymenostomatida | 0.536 | -0.098 | 0.250 | 0.225 | 0.1508 | 0.133 | 0.991 | 0.207 |
| Paramecium | 2.36 | 1.71 | 2.08 | 2.00 | 0.167 | 0.133 | 0.991 | 0.207 |
| Pseudocohnilembus | 1.46 | 0.82 | 1.14 | 1.06 | 0.165 | 0.133 | 0.991 | 0.207 |
| UC_Oligohymenophoera | 2.22 | 1.62 | 1.97 | 1.90 | 0.169 | 0.134 | 0.991 | 0.208 |
| Halteria | 1.82 | 1.08 | 1.38 | 1.41 | 0.160 | 0.133 | 0.991 | 0.207 |
| Stylonichia | 2.39 | 1.73 | 2.14 | 2.07 | 0.182 | 0.134 | 0.991 | 0.208 |
| UC_Sporadotrichia | 1.24 | 0.54 | 0.88 | 0.90 | 0.191 | 0.149 | 0.991 | 0.207 |
| UC_Ciliophora | 3.43 | 2.75 | 3.09 | 3.07 | 0.182 | 0.134 | 0.991 | 0.207 |

RA_clr: relative abundance CLR-transformed; HF: Holstein-fresian; BS: brown Swiss; CTR: control; CHI: chitosan; SEM: standard error of the mean; T: treatment; Adjust P-value: adjusted P-value using False Discovery Rate; UC: unclassified.
